# Supplementary material for: ProteinShader: illustrative rendering of macromolecules
Source: BMC Struct Biol. 2009 Mar 30;9:19. doi: 10.1186/1472-6807-9-19 (PMC2672931; doi:10.1186/1472-6807-9-19)
Supplement: Additional file 1 — ProteinShader program without source code. This compressed file contains the complete ProteinShader program including associated libraries, but no source code. A README.txt file gives an overview of the ProteinShader distribution, and the index.html file in the help subdirectory has directions on getting started with the program as well as a set of tutorials. [file 1472-6807-9-19-S1.zip › ProteinShader-beta-0_9_4-binary/help/api/org/proteinshader/gui/class-use/Mediator.html]

Uses of Interface org.proteinshader.gui.Mediator (ProteinShader API)


|  |  |  |
| --- | --- | --- |
| **Overview** \| **Package** \| **Class** \| **Use** \| **Tree** \| **Deprecated** \| **Index** \| **Help** | |  |
| PREV   NEXT | **FRAMES**    **NO FRAMES**     **All Classes** |  |


---


## **Uses of Interface org.proteinshader.gui.Mediator**

| Packages that use Mediator | |
| --- | --- |
| **org.proteinshader.gui** | Holds all of the Swing GUI components and their associated listeners, including class Renderer, which is registered as a listener for the GLCanvas object that is used a drawing surface. |
| **org.proteinshader.gui.components.controlpanel** | Holds the JPanels used in the right-side retractable control panel of the GUI. |
| **org.proteinshader.gui.components.menubar** | Holds the JMenus used in menu bar above the canvas. |
| **org.proteinshader.gui.listeners** | Holds the listener classes for the gui. |
| **org.proteinshader.gui.listeners.controlpanel** | Holds the listeners for components used in the JPanels of the right-side retractable control panel of the GUI. |
| **org.proteinshader.gui.listeners.menubar** | Holds the listeners for the items used in the JMenus of the menu bar above the canvas. |
| **org.proteinshader.gui.viewing** | Holds important helper classes that the Renderer uses to control the view: Camera, Rotation, Lighting, *etc*. |

Uses of Mediator in org.proteinshader.gui

| Classes in org.proteinshader.gui that implement Mediator | |
| --- | --- |
| `class` | `MediatorImpl`             Serves as the central mediator central object that allows communication between objects of the gui, the data structure, and the renderer. |

Constructors in org.proteinshader.gui with parameters of type Mediator

`Renderer(Mediator mediator)`
  
          Constructs a Renderer.

Uses of Mediator in org.proteinshader.gui.components.controlpanel

Constructors in org.proteinshader.gui.components.controlpanel with parameters of type Mediator

`AntialiasingPanel(Mediator mediator,
Frame dialogOwner)`
  
          Constructs an AntialiasingPanel.

`AtomColorPanel(Mediator mediator,
Frame dialogOwner,
RadioPanel radioPanel)`
  
          Constructs a ColorPanel.

`AtomScalePanel(Mediator mediator,
Frame dialogOwner,
RadioPanel radioPanel)`
  
          Constructs a AtomScalePanel.

`AtomVisibilityPanel(Mediator mediator,
Frame dialogOwner,
RadioPanel radioPanel)`
  
          Constructs a VisibilityPanel.

`CartoonColorPanel(Mediator mediator,
Frame dialogOwner,
RadioPanel radioPanel)`
  
          Constructs a CartoonColorPanel.

`CartoonSideChainsPanel(Mediator mediator,
Frame dialogOwner,
RadioPanel radioPanel)`
  
          Constructs a CartoonSideChainsPanel.

`CartoonVisibilityPanel(Mediator mediator,
Frame dialogOwner,
RadioPanel radioPanel)`
  
          Constructs a CartoonVisibilityPanel.

`ColorPanel(Mediator mediator,
Frame dialogOwner,
RadioPanel radioPanel,
String default1Name,
String default2Name)`
  
          Constructs a ColorPanel.

`ControlPanel(Mediator mediator,
Frame dialogOwner)`
  
          Constructs a ControlPanel.

`DecorationsPanel(Mediator mediator,
Frame dialogOwner,
RadioPanel radioPanel)`
  
          Constructs a DecorationsPanel.

`ModifierPanel(Mediator mediator,
Frame dialogOwner)`
  
          Constructs a ModifierPanel.

`MotionPanel(Mediator mediator,
Frame dialogOwner)`
  
          Constructs a MotionPanel.

`OptimizePanel(Mediator mediator,
Frame dialogOwner)`
  
          Constructs an OptimizePanel.

`RadioPanel(Mediator mediator,
Frame dialogOwner,
RadioPanel.Type type)`
  
          Constructs a RadioPanel.

`SelectorPanel(Mediator mediator,
Frame dialogOwner)`
  
          Constructs a SelectorPanel.

`VisibilityPanel(Mediator mediator,
Frame dialogOwner,
RadioPanel radioPanel)`
  
          Constructs a VisibilityPanel.

Uses of Mediator in org.proteinshader.gui.components.menubar

Constructors in org.proteinshader.gui.components.menubar with parameters of type Mediator

`BackgroundMenu(Mediator mediator,
Frame dialogOwner)`
  
          Constructs a BackgroundMenu.

`FileMenu(Mediator mediator)`
  
          Constructs a FileMenu with menu items "File" and "Quit", and an "Export
Image" submenu for saving PNG and JPEG files.

`HelpMenu(Mediator mediator,
Frame dialogOwner)`
  
          Constructs a HelpMenu.

`MainMenuBar(Mediator mediator)`
  
          Constructs a ProteinShaderMenuBar.

`OrientationMenu(Mediator mediator)`
  
          Constructs a OrientationMenu.

`StyleMenu(Mediator mediator)`
  
          Constructs a StyleMenu.

`ToolsMenu(Mediator mediator)`
  
          Constructs a ToolsMenu.

`VisibilityMenu(Mediator mediator)`
  
          Constructs a VisibilityMenu.

Uses of Mediator in org.proteinshader.gui.listeners

Constructors in org.proteinshader.gui.listeners with parameters of type Mediator

`CanvasKeyListener(Mediator mediator,
Renderer renderer,
GLCanvas canvas)`
  
          Constructs a CanvasKeyListener.

`WindowListenerFactory(Mediator mediator)`
  
          Constructs a WindowListenerFactory.

Uses of Mediator in org.proteinshader.gui.listeners.controlpanel

Constructors in org.proteinshader.gui.listeners.controlpanel with parameters of type Mediator

`AntialiasingPanelListenerFactory(Mediator mediator,
AntialiasingPanel antialiasingPanel)`
  
          Constructs a AntialiasingPanelListenerFactory.

`CartoonSideChainsPanelListenerFactory(Mediator mediator,
CartoonSideChainsPanel sideChainsPanel)`
  
          Constructs a CartoonSideChainsPanelListenerFactory.

`ColorPanelListenerFactory(Mediator mediator,
ColorPanel colorPanel)`
  
          Constructs a ColorPanelListenerFactory.

`DecorationsPanelListenerFactory(Mediator mediator,
DecorationsPanel panel)`
  
          Constructs a DecorationsPanelListenerFactory.

`MotionPanelListenerFactory(Mediator mediator,
MotionPanel motionPanel)`
  
          Constructs a MotionPanelListenerFactory.

`OptimizePanelListenerFactory(Mediator mediator,
OptimizePanel optimizePanel)`
  
          Constructs a OptimizePanelListenerFactory.

`ScalePanelListenerFactory(Mediator mediator,
AtomScalePanel scalePanel)`
  
          Constructs a ScalePanelListenerFactory.

`SelectorPanelListenerFactory(Mediator mediator,
SelectorPanel selector)`
  
          Constructs a SelectorPanelListenerFactory.

`VisibilityPanelListenerFactory(Mediator mediator,
VisibilityPanel visibilityPanel)`
  
          Constructs a VisibilityPanelListenerFactory.

Uses of Mediator in org.proteinshader.gui.listeners.menubar

Constructors in org.proteinshader.gui.listeners.menubar with parameters of type Mediator

`BackgroundMenuListenerFactory(Mediator mediator,
BackgroundMenu backgroundMenu)`
  
          Constructs a BackgroundMenuListenerFactory.

`FileMenuListenerFactory(Mediator mediator)`
  
          Constructs a FileMenuListenerFactory.

`HelpMenuListenerFactory(Mediator mediator,
HelpMenu helpMenu)`
  
          Constructs a HelpMenuListenerFactory.

`OrientationMenuListenerFactory(Mediator mediator)`
  
          Constructs a OrientationMenuListenerFactory.

`StyleMenuListenerFactory(Mediator mediator)`
  
          Constructs a StyleMenuListenerFactory.

`ToolsMenuListenerFactory(Mediator mediator)`
  
          Constructs a ToolsMenuListenerFactory.

`VisibilityMenuListenerFactory(Mediator mediator)`
  
          Constructs a VisibilityMenuListenerFactory.

Uses of Mediator in org.proteinshader.gui.viewing

Constructors in org.proteinshader.gui.viewing with parameters of type Mediator

`Rotation(Mediator mediator,
FpsLabel fpsLabel)`
  
          Constructs a Renderer.

---


|  |  |  |
| --- | --- | --- |
| **Overview** \| **Package** \| **Class** \| **Use** \| **Tree** \| **Deprecated** \| **Index** \| **Help** | |  |
| PREV   NEXT | **FRAMES**    **NO FRAMES**     **All Classes** |  |


---

# *Copyright © 2007-2008*
